# Integration of somatosensory and motor-related information in the auditory system

**DOI:** 10.3389/fnins.2022.1010211

**Published:** 2022-10-18

**Authors:** Michael Lohse, Paul Zimmer-Harwood, Johannes C. Dahmen, Andrew J. King

**Affiliations:** Department of Physiology, Anatomy & Genetics, University of Oxford, Oxford, United Kingdom

**Keywords:** auditory, somatosensory, multisensory integration, movement, cortex, thalamus, midbrain, perception

## Abstract

An ability to integrate information provided by different sensory modalities is a fundamental feature of neurons in many brain areas. Because visual and auditory inputs often originate from the same external object, which may be located some distance away from the observer, the synthesis of these cues can improve localization accuracy and speed up behavioral responses. By contrast, multisensory interactions occurring close to the body typically involve a combination of tactile stimuli with other sensory modalities. Moreover, most activities involving active touch generate sound, indicating that stimuli in these modalities are frequently experienced together. In this review, we examine the basis for determining sound-source distance and the contribution of auditory inputs to the neural encoding of space around the body. We then consider the perceptual consequences of combining auditory and tactile inputs in humans and discuss recent evidence from animal studies demonstrating how cortical and subcortical areas work together to mediate communication between these senses. This research has shown that somatosensory inputs interface with and modulate sound processing at multiple levels of the auditory pathway, from the cochlear nucleus in the brainstem to the cortex. Circuits involving inputs from the primary somatosensory cortex to the auditory midbrain have been identified that mediate suppressive effects of whisker stimulation on auditory thalamocortical processing, providing a possible basis for prioritizing the processing of tactile cues from nearby objects. Close links also exist between audition and movement, and auditory responses are typically suppressed by locomotion and other actions. These movement-related signals are thought to cancel out self-generated sounds, but they may also affect auditory responses via the associated somatosensory stimulation or as a result of changes in brain state. Together, these studies highlight the importance of considering both multisensory context and movement-related activity in order to understand how the auditory cortex operates during natural behaviors, paving the way for future work to investigate auditory-somatosensory interactions in more ecological situations.

## Introduction

Vision and hearing provide the primary source of information about distant objects and events that may be too far away to engage the other senses. Furthermore, visual and auditory cues arising from the same source are often complementary and integrated by the brain in ways that shape our perception of the information provided by the source, including its location, timing and identity (see Opoku-Baah et al., 2021 for a recent review). The visual and auditory systems are equally important for processing inputs from nearby sources, such as when we are reading text on or listening to our mobile phones. Within this proximal region of space, other sensory modalities come more into play, which therefore need to be considered too if we are to understand the neural basis for perception and behaviors that are guided by sensory cues close to the head and body.

In this review, we examine how the processing of auditory inputs is influenced by other sensory modalities near the head, focusing primarily on interactions with the somatosensory system. We start by considering how the sound field changes with distance from the source and the implications of this for perceiving nearby auditory objects. We examine the evidence for sensitivity to auditory target distance, including the contribution of auditory inputs to the encoding of space close to the body, before looking at the way auditory and somatosensory signals interact to influence perception and neuronal responses, particularly within the auditory regions of the brain. Most studies have examined these crossmodal interactions by presenting sounds whilst applying vibrotactile stimuli to the skin, but proprioceptive signals arising from changes in body orientation also need to be considered to understand the full extent to which somatosensory signals are integrated with auditory processing. Finally, because a range of actions—including exploratory movements, reaching and grasping, as well as avoidance responses—can be elicited by nearby sensory stimuli, we examine the impact of motor-related activity on auditory processing, highlighting some of the similarities between the effects of movement, brain state and somatosensory cues on the auditory system.

## Sound propagation and distance cues

**FIGURE 1:**
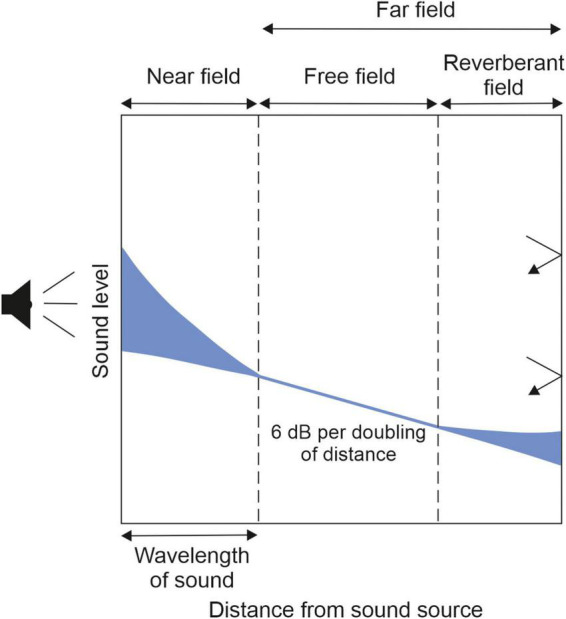
At locations near a sound source **(left)**, or near the walls of a reverberant room **(right)**, sound levels are variable and do not follow the normal, free-field rule of decrease with the inverse square of distance. The blue areas on the graph represent the variable influences of the near field and reverberation. Adapted from Moore and King (1999).

Sound waves behave differently as they propagate away from the source (Figure 1; Moore and King, 1999). Within the near field, the region of the sound field closest to the source, a complex relationship exists between sound pressure level and distance as some of the energy circulates without propagating. At a distance corresponding to roughly one wavelength, the far field begins, which can be subdivided into a free-field region where the sound pressure level follows the inverse square law, decreasing by 6 dB with each doubling of the distance from the source, and a reverberant field, where reflections from the walls and other surfaces within the room result in delayed and distorted versions of the direct sound. These properties give rise to several cues that help the brain to encode sound sources as they vary with distance from the listener. They include changes in level, frequency composition (with high frequencies scattered more at longer distances), the ratio of direct-to-reverberant sound energy (which declines with increasing distance), and, for sounds that are sufficiently close to fall within the acoustic near field, variations in interaural level differences on each side of the listener (Brungart and Rabinowitz, 1999; Kolarik et al., 2016).

## Sensitivity to sound-source distance

Most studies of auditory distance perception in humans have focused on the far-field region of space (sometimes using virtual acoustic space stimuli to simulate distances of around a meter or more from the listener’s ears), where this relies principally on estimates of the level of the sound and the difference in level between the direct and reverberant sound (Bronkhorst and Houtgast, 1999; Zahorik, 2002; Kolarik et al., 2013). The relative contribution of these two cues depends on the type of sound used and its direction (Zahorik, 2002), and changes with distance and room reverberation time (Kolarik et al., 2013). Of course, sound level provides a useful absolute distance cue only if the source has a fixed level and a number of other factors, including other sensory cues and body orientation, have been shown to affect auditory distance perception (Harris et al., 2015; Kolarik et al., 2016). In particular, auditory distance judgments can be influenced by the more accurate and reliable spatial information provided by visual stimuli (Loomis et al., 1998; Zahorik, 2001; Anderson and Zahorik, 2014; Kolarik et al., 2016).

Behavioral studies in humans (Brungart, 1999; Zahorik, 2002; Kopčo and Shinn-Cunningham, 2011) and rabbits (Kuwada et al., 2015) have also demonstrated distance sensitivity for sound sources in close proximity to the head. In the absence of sound-level cues, a comparison of the levels of direct and reverberant sound energy appears to play the dominant role (Kopčo and Shinn-Cunningham, 2011). However, if there are no room reflections, as in anechoic conditions, it is likely that interaural level differences in low-frequency sounds (< 3 kHz) are used to judge the distance of nearby lateral sources (Brungart, 1999). Neurophysiological evidence for level-independent sensitivity to the distance of nearby sound sources has been obtained using fMRI in human non-primary posterior auditory cortex, with these findings again suggesting that this is based primarily on a comparison of the levels of direct and reverberant sounds (Kopěo et al., 2012; Kopco et al., 2020). Since amplitude modulation depth changes with the ratio of direct-to-reverberant sound energy—and therefore the source distance—it has been suggested that the sensitivity of neurons in the inferior colliculus (IC) to the depth of amplitude modulation might provide a basis for using this cue to represent sound-source distance (Kim et al., 2015).

## Auditory contributions to the representation of peripersonal space

Sensitivity to sounds close to the body, and particularly for looming sounds that are likely to be indicative of an approaching object (Seifritz et al., 2002; Maier et al., 2008), contributes to the representation in the brain of peripersonal space, the region of space within our immediate reach, where exteroceptive information from the eyes and ears interacts with somatosensory inputs. Multisensory processing in peripersonal space provides information about the position of the body in the environment and helps to localize nearby objects (Serino, 2019).

The processing of multisensory signals in peripersonal space has been studied extensively within a frontoparietal network of the primate brain (reviewed by Serino, 2019). Neurons in these areas respond both to tactile stimuli on specific parts of the body and to visual and/or auditory stimuli in close proximity to the body. For example, acoustically-responsive neurons recorded in the ventral premotor cortex of awake monkeys were found to respond more strongly when broadband sounds were placed closer to the head, often independently of stimulus level (Graziano et al., 1999). The majority of these neurons also responded to visual and tactile stimulation, and are thought to represent sensory information that is used to guide reaching or avoidance responses (Graziano and Gross, 1998). Furthermore, behavioral (Occelli et al., 2011; Teneggi et al., 2013) and electrophysiological (Bernasconi et al., 2018) studies in humans have reported that sounds are more likely to influence tactile processing when they are presented in close proximity to the head than further away.

While this work highlights the importance of detecting and responding to nearby sounds, particularly those looming toward the body, the methods used for investigating sensitivity to sound-source distance in many studies of peripersonal space are limited in terms of the range of sound-source locations tested and by the lack of attention to the way sound properties change with distance within the near field. Indeed, whether auditory-tactile peripersonal space exists at all has been questioned (Holmes et al., 2020), and the frontoparietal cortical areas in macaques and humans that have been the focus of peripersonal space research are dominated by visual and somatosensory, rather than auditory, inputs (Macaluso and Maravita, 2010).

External objects generating auditory (or visual) signals that move closer to the head and body can signify approaching danger, which therefore requires a rapid response (Graziano and Cooke, 2006). However, integration of tactile information with other sensory modalities is not limited to those situations. Palpation and manipulation of objects in peripersonal space, using the hands, feet, mouth or—especially in rodents (Sofroniew and Svoboda, 2015)—*via* exploratory movements of mechanosensitive whiskers, will often generate sound. This may occur as a direct result of the haptic interactions with the object, but sounds can also be produced by locomotory or other accompanying movements required to execute these actions. There are therefore many situations where auditory and somatosensory signals are present at the same time and it is important to understand how they are combined and integrated in the brain.

## Perceptual consequences of interactions between auditory and somatosensory inputs

**FIGURE 2:**
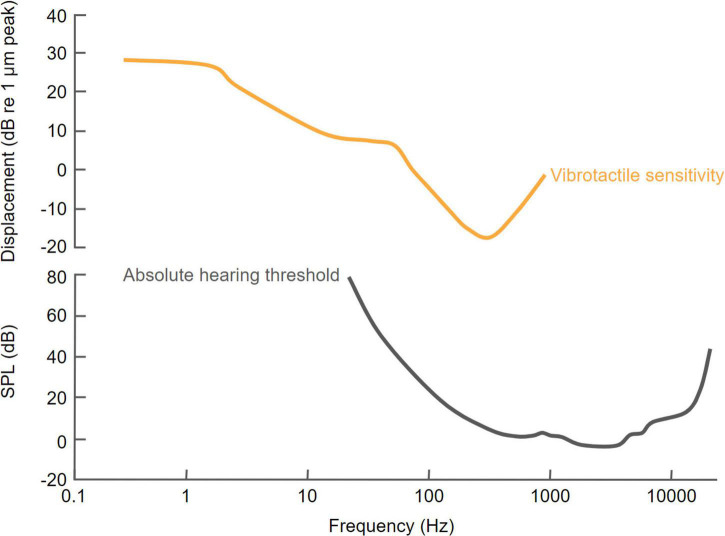
Comparison of human sensitivity to vibrotactile and acoustic stimuli as a function of the frequency of stimulation. The vibrotactile sensitivity curve is based on Gescheider et al. (2002) and the hearing threshold curve is based on International Standard ISO 389-7: 2003(E) (Reference threshold of hearing under free-field and diffuse-field listening conditions).

Although most perceptual studies of multisensory integration have focused on vision and audition (Opoku-Baah et al., 2021), interactions can occur across all sensory modalities and there is growing evidence for close links between auditory and tactile processing. Over the past 20 years, numerous studies have explored the effects of auditory-tactile interactions on human perception. In fact, similarities between audition and touch were recognized much earlier (Von Békésy, 1959), including the fact that both senses are based on the detection of frequency-dependent mechanical displacements. Sensitivity to the frequency of mechanical vibrations in each modality, which overlap in a low-frequency range that extends up to about 1,000 Hz, is an important factor in determining how they interact (Soto-Faraco and Deco, 2009; Figure 2).

Simultaneous audio-tactile stimulation can improve reaction times (Sperdin et al., 2009; Godenzini et al., 2021) and stimulus detection (Schürmann et al., 2004; Gillmeister and Eimer, 2007; Ro et al., 2009), and increase perceived loudness (Gillmeister and Eimer, 2007; Yarrow et al., 2008). These interactions have been shown to occur in a frequency-dependent manner (Ro et al., 2009; Tajadura-Jiménez et al., 2009; Wilson et al., 2010a,b), with the largest perceptual improvements reported when the frequencies of the auditory and vibrotactile stimuli are closely matched (Ro et al., 2009; Wilson et al., 2010b). More generally, auditory-tactile interactions are particularly prominent in the temporal domain (Occelli et al., 2011) and therefore play a fundamental role in the way individuals interact with their environments, as well as in the production and perception of music and speech (Ito et al., 2009; Occelli et al., 2011; Keough et al., 2019).

There is a growing body of evidence from studies in human participants that the auditory and somatosensory systems can reciprocally bias each other (Soto-Faraco and Deco, 2009; Occelli et al., 2011; Villanueva and Zampini, 2018). Perceptual interactions between these modalities are, however, to some extent task dependent and asymmetric. This is often explored by asking participants to attend to one modality while ignoring distractor stimuli in the other modality. For example, performance on a tactile frequency-discrimination task is impaired by task-irrelevant auditory distractors, so long as the auditory stimulus is of a similar frequency to the attended tactile stimulus, whereas judgments of the intensity of the tactile stimulus are unaffected (Yau et al., 2009). By contrast, the presence of a tactile distractor can bias both the perceived frequency and intensity of an auditory stimulus (Yau et al., 2010). Crossmodal adaptation paradigms, in which the different stimuli are separated in time, have shown that an auditory adaptor can also influence the perception of vibrotactile frequency but not intensity, with this interaction again limited to overlapping frequencies in each modality (Crommett et al., 2017). The impact of stimulus frequency in integrating audition and touch is highlighted by the parchment-skin illusion. Increasing over headphones the high-frequency components of sound accompanied by the motion of rubbing the hands together leads to subjects reporting a drier or rougher sensation of their skin (Jousmäki and Hari, 1998).

Psychophysical evidence for frequency-specific audio-tactile interactions has been observed for a wide range of repetition rates, extending down into the flutter range (< 50 Hz) (Convento et al., 2019), where the stimuli are perceived as a series of individual pulses rather than a vibratory hum, and in the perception of frequency sweeps (Crommett et al., 2019). This is likely to be important for sensing objects that generate correlated tactile and auditory signals, as in the aforementioned example of texture perception (Lederman, 1979). These findings indicate that auditory and tactile frequency representations are integrated in the brain. In humans, cortical regions exhibiting sensitivity to both tactile and auditory frequency information appear to be sparsely distributed within modality-specific areas in the parietal and temporal lobes (Rahman et al., 2020), while interactions between these modalities in mouse somatosensory cortical areas have been found to depend on the frequency of tactile stimulation (Zhang et al., 2020).

**FIGURE 3:**
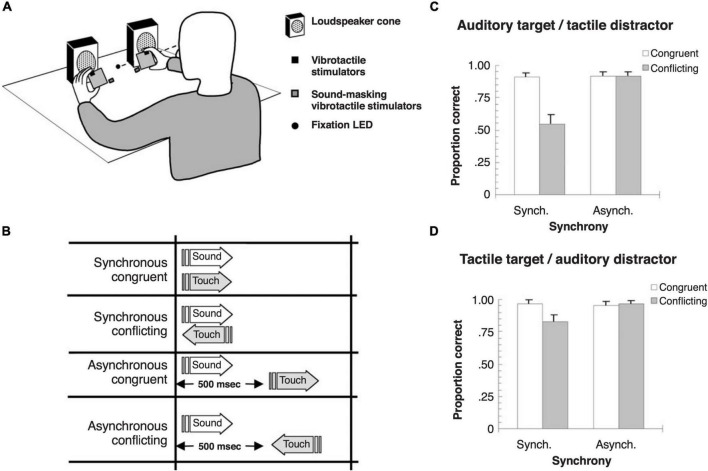
Crossmodal capture of the perception of apparent motion direction with auditory and tactile stimuli in humans. **(A)** Schematic of the experimental setup. Tactile stimuli were delivered by hand-held vibrating foam cubes, and auditory stimuli were presented from two loudspeakers. Participants were asked to evaluate whether auditory or tactile target stimuli were moving to the right or left while ignoring stimulation in the other distractor modality. **(B)** The direction of apparent motion for the two modalities was either congruent or conflicting, with the stimuli presented either synchronously or asynchronously, with a 500 ms delay between the target and distractor. **(C)** Effects of a tactile motion distractor on the accuracy of auditory motion direction judgments. Note that task-irrelevant tactile motion in the opposing direction impaired the ability of participants to detect the correct sound movement direction, but only when the stimuli were presented synchronously. **(D)** Effects of an auditory motion distractor on the accuracy of tactile motion direction judgments. A smaller crossmodal modulatory effect was observed when the tactile stimulus was the target and the auditory stimulus the distractor. Adapted with permission from Soto-Faraco et al. (2004).

In the spatial domain, where vision is normally the dominant sense, behavioral and electrophysiological measurements in humans have shown that similar crossmodal interactions take place between auditory and somatosensory stimuli irrespective of their locations (Murray et al., 2005). Nevertheless, several studies have demonstrated that auditory spatial responses can be biased by tactile or proprioceptive inputs (Pick et al., 1969; Caclin et al., 2002; Sanabria et al., 2005; Bruns and Röder, 2010). Just as exposure to a consistent spatial mismatch between auditory and visual stimuli shifts auditory localization judgments that persist for a few minutes when sounds are subsequently presented alone (Radeau and Bertelson, 1974; Recanzone, 1998), an audio-tactile ventriloquism aftereffect exists, suggesting that auditory space is continually recalibrated to compensate for spatial disparities between these modalities too (Bruns et al., 2011). Somatosensory capture of auditory motion perception has also been demonstrated (Soto-Faraco et al., 2004; Figure 3). Tactile distractors moving in a conflicting direction disrupt auditory motion perception (Figure 3C), whereas auditory motion distractors have a smaller effect on the apparent direction of motion of tactile stimuli (Figure 3D).

Different sensory cues appear to be combined and integrated in a statistically optimal way according to their relative reliability (Ernst and Bülthoff, 2004). While this can explain why certain sensory modalities appear to dominate and capture specific aspects of perception in other modalities, it is also important to take differences between species and their habitats into account. Thus, in mice, which are nocturnal animals, the weighting between vision and audition is reversed, with audition dominating vision when perceptual decisions are made in the presence of conflicting visual and auditory information (Song et al., 2017), suggesting that “sensory hierarchies” can be species as well as task specific. By the same token, given their reliance on whisker movements for exploratory behavior and tactile discrimination (Sofroniew and Svoboda, 2015), there is evidence that rodents may place even more weight on the somatosensory system when combining inputs across different sensory modalities (Rao et al., 2014). This highlights the importance of considering the natural ecology of the species in question when considering the functional consequences and neural underpinnings of multisensory integration in the brain.

## Interactions between auditory and somatosensory inputs in the brain

Given the extensive evidence for the effects of combining auditory and tactile cues on human perception, it is not surprising that the majority of studies exploring the neural substrates for these multisensory interactions in both humans and other species have focused on the cerebral cortex. In addition to the higher-level cortical association areas implicated in the representation of peripersonal space, multisensory signals have been found to converge throughout the cortex, including in auditory, somatosensory and visual early cortical areas (reviewed in Ghazanfar and Schroeder, 2006; Meijer et al., 2019). Thus, responses to auditory stimulation in somatosensory cortical areas have been reported in both human fMRI (Liang et al., 2013; Pérez-Bellido et al., 2018; Rahman et al., 2020) and magnetoencephalography studies (Lütkenhöner et al., 2002), as well as in single-neuron measurements in monkeys (Zhou and Fuster, 2004) and mice (Carvell and Simons, 1986; Zhang et al., 2020; Godenzini et al., 2021). More attention has been paid, however, to the way that other sensory modalities affect activity in the auditory cortex, with somatosensory influences demonstrated using a range of methods and in several different species (Foxe et al., 2002; Fu et al., 2003; Brosch et al., 2005; Kayser et al., 2005; Schürmann et al., 2006; Lakatos et al., 2007; Lemus et al., 2010; Iurilli et al., 2012; Nordmark et al., 2012; Hoefer et al., 2013; Rao et al., 2014; Lohse et al., 2021).

While most studies of multisensory processing have shown that sound-evoked responses in the auditory cortex are modulated by somatosensory or visual stimuli, there are several reports in different animal species that some neurons in the auditory cortex can be driven by other sensory modalities (Fu et al., 2003; Brosch et al., 2005; Bizley et al., 2007; Lemus et al., 2010; Meredith and Allman, 2015; Morrill and Hasenstaub, 2018). The functional significance of responses evoked by the “non-principal” modality in auditory or other sensory cortical areas is unknown. Lemus et al. (2010) reported that neurons exist in macaque monkey primary auditory cortex (A1) that respond to somatosensory stimulation, but were unable to decode the identity of the tactile stimulus from the activity of these neurons. There is some indication that responses in auditory cortex to visual and somatosensory stimuli can arise as a result of the behavioral procedure used to train monkeys over long periods in auditory tasks (Brosch et al., 2005). However, this does not explain the presence of these neurons in many other studies, where they have been most commonly reported under anesthesia or in awake animals that were not engaged in a sensory task.

**FIGURE 4:**
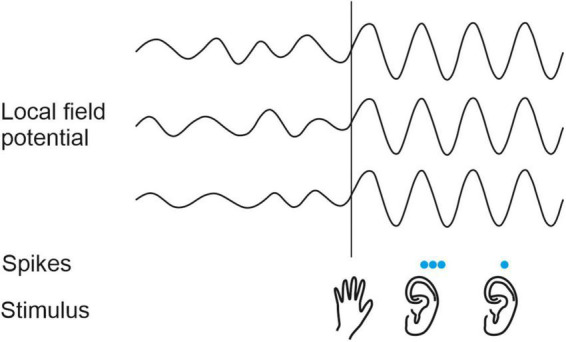
Modulatory influence of somatosensory stimuli on the auditory cortex in non-human primates. A somatosensory stimulus (hand, **bottom row**) resets the phase of the local field potential (LFP) oscillations in auditory cortex **(top traces)**. As a result, auditory stimuli (ears, **bottom row**) are more likely to elicit spikes (blue dots, **middle row**) when they arrive during peaks than during troughs in the ongoing LFP. Based on Lakatos et al. (2007) and Kayser (2009).

On the basis of the changes induced in sound-evoked response properties, the modulatory influence of vision on the auditory cortex has been implicated in various perceptual phenomena, including visual enhancement of speech perception (Schroeder et al., 2008), sound localization (Bizley and King, 2008) and auditory scene analysis (Atilgan et al., 2018), as well as the ventriloquist illusion (Bonath et al., 2014; Zierul et al., 2017). Tactile (and visual) inputs have been shown to reset the phase of ongoing neuronal oscillations in the auditory cortex, effectively amplifying the response to auditory events that are aligned with the peaks in the oscillations (Lakatos et al., 2007; Kayser et al., 2008; Kayser, 2009; Figure 4). This in turn offers a potential explanation for the effects of somatosensory stimulation on sound intensity perception that were described in the previous section.

A close interaction between what is heard and felt is an essential aspect of playing a musical instrument, and studies of musical training have provided unique insights into the plasticity of multisensory integration at a perceptual level and demonstrated that physiological and anatomical changes take place in the relevant cortical areas (Münte et al., 2002; Herholz and Zatorre, 2012). For example, compared to non-musicians, trumpet players display significantly increased cortical signal strength for combined auditory-somatosensory stimuli exclusively for tactile stimulation of their lips (Schulz et al., 2003). Musicians have also been found to react faster to tactile and non-musical auditory stimuli presented either separately or together (Landry and Champoux, 2017), are less affected by the audio-tactile flash illusion—where presentation of one tactile stimulus with multiple task-irrelevant tones normally leads to the perception of more than one touch (Landry et al., 2017), and are more sensitive to audio-tactile incongruencies than non-musicians (Kuchenbuch et al., 2014). In fact, musical training also appears to narrow the temporal integration window for binding auditory and visual signals (Petrini et al., 2009), which is seen for music, but not speech (Lee and Noppeney, 2011). Changes in multisensory processing in the auditory cortex are not only limited to professional musicians (Pantev et al., 2015), since auditory-somatosensory training in non-musicians can result in greater cortical plasticity than auditory-only training (Pantev et al., 2009).

## Crossmodal suppression of cortical activity

The modulatory influence of somatosensory inputs on auditory cortical responses ranges from mostly facilitatory effects in monkeys (Fu et al., 2003; Kayser et al., 2005; Lakatos et al., 2007) to predominantly suppressive interactions in ferrets (Meredith and Allman, 2015), cats (Khorevin and Shelest, 1998) and rodents (Iurilli et al., 2012; Rao et al., 2014; Lohse et al., 2021), at least in the primary or core auditory areas. This may indicate that somatosensory stimuli that are more salient behaviorally can downregulate responses to potentially distracting sounds that are experienced at the same time. The finding by Rao et al. (2014) that facial touch during social interactions with conspecifics inhibits activity in A1 of rats supports this possibility, and provides a rare example of multisensory processing in a natural ecological context. Similar conclusions about the interplay between these modalities have also been drawn in studies demonstrating suppression of auditory responses in somatosensory cortical areas (Gobbelé et al., 2003; Zhang et al., 2020).

**FIGURE 5:**
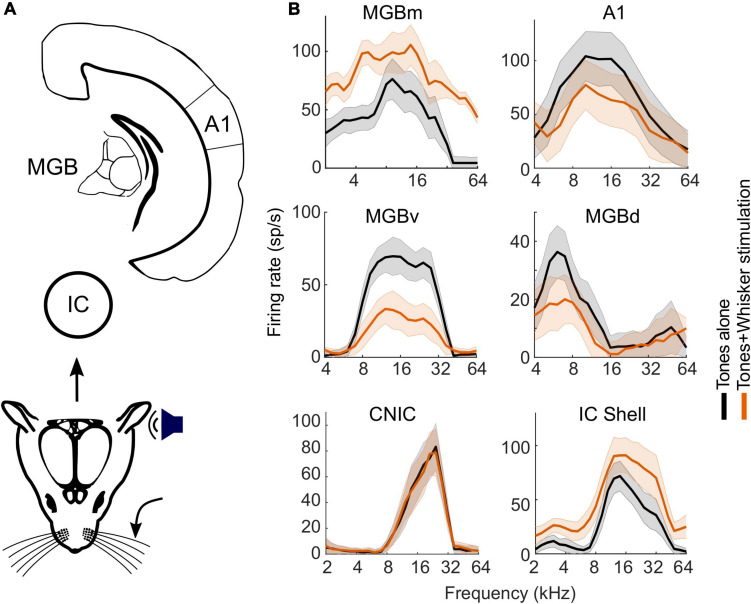
Somatosensory influence on spectral tuning in the auditory midbrain, thalamus, and cortex of mice. **(A)** Schematic of the auditory pathway from midbrain to cortex. **(B)** Frequency response profiles from representative neurons (electrophysiologically recorded units) with and without concurrent whisker stimulation. Note the lack of effect of whisker stimulation in the CNIC, whereas an additive facilitatory effect was observed in the IC shell and in MGBm, and divisive suppression was found in MGBv, MGBd and in A1. Recordings in the IC were obtained in both anesthetized and awake mice; no differences were observed in the effects of whisker stimulation on auditory responses according to whether the mice were anesthetized or not. A1, primary auditory cortex; IC, inferior colliculus; CNIC, central nucleus of the IC; MGB, medial geniculate body; MGBd, MGBm, MGBv, dorsal, medial and ventral divisions of the MGB. Adapted from Lohse et al. (2021).

A recent study by Lohse et al. (2021) showed that these crossmodal interactions in mouse A1 are divisive in nature with passive whisker stimulation suppressing auditory responses primarily around the preferred sound frequency of the neurons, as shown by the changes in the frequency response profiles recorded at different levels of the auditory pathway (Figure 5). Divisive normalization is regarded as one of the hallmarks of multisensory integration, and has also been implicated in audio-tactile interactions in the mouse somatosensory cortex (Zhang et al., 2020) and in the way neuronal responses in other brain areas are determined by the efficacy and spatial relationship of the individual stimuli (Ohshiro et al., 2011). This is likely to represent a “canonical operation” performed by sensory neurons, which could be implemented by various biophysical mechanisms, many of which involve some form of inhibition (Carandini and Heeger, 2011). In this regard, it is interesting that crossmodal divisive scaling by non-driving somatosensory inputs is also found in mouse auditory thalamocortical neurons (Lohse et al., 2021), which, at least in rats, lack local recurrent connectivity (Bartlett and Smith, 1999).

From a functional perspective, divisive scaling of A1 responses has also been linked to behavioral improvements in frequency discrimination at the expense of sound detection performance (Guo et al., 2017). Related to this is the finding that suppression of auditory cortical responses by visual inputs is associated with an increase in response reliability and in the amount of stimulus-related information transmitted (Bizley et al., 2007; Kayser et al., 2010). Although more research is needed in behaving animals, it is possible that while tactile inputs reduce auditory cortical activity, potentially prioritizing stimuli that touch the whiskers or other parts of the body, they may actually serve to enhance auditory acuity.

## Neural circuits underlying auditory-somatosensory interactions

**FIGURE 6:**
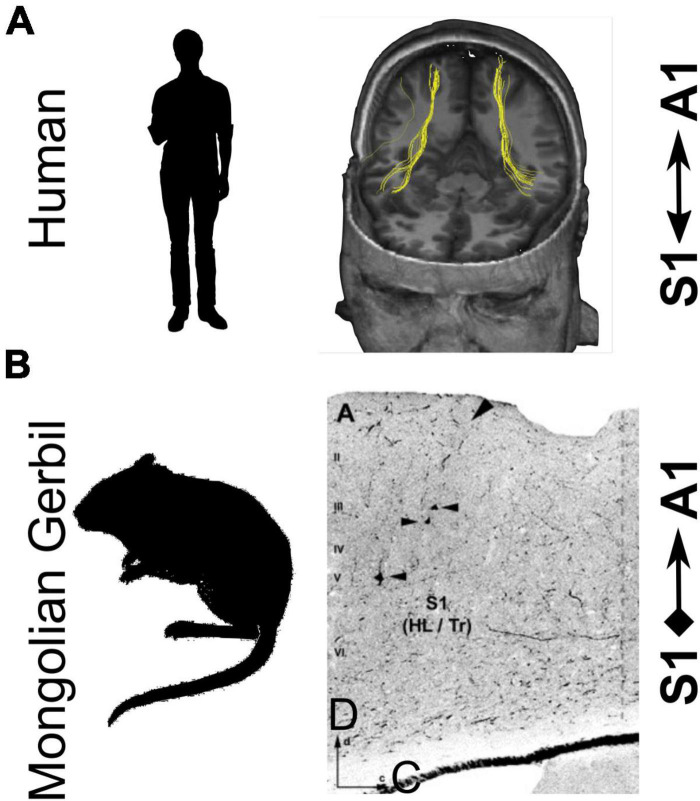
Direct connectivity between the primary somatosensory cortex (S1) and primary auditory cortex (A1) in humans and rodents. **(A)** Fiber tracts connecting S1 and A1 in human cortex demonstrated using diffusion tensor imaging with deterministic tractography. **(B)** Direct projections from S1 to A1 in Mongolian gerbils were demonstrated by injecting fluorescein-labeled dextran into A1. Arrowheads indicate retrogradely-labeled cells in S1. HL/Tr, hindlimb/trunk regions; D, dorsal; C, caudal. Panel **(A)** is adapted with permission from Ro et al. (2013). Panel **(B)** is adapted with permission from Budinger et al. (2006).

An important question that has implications for both the nature of the neural circuitry involved and the role of multisensory interactions in goal-directed behavior is where they take place in the brain. Convergence of inputs from different modalities in early cortical areas could arise from direct connections between those areas, feedback from higher-level associations areas, or be inherited from subcortical inputs (Driver and Noesselt, 2008; Cappe et al., 2012). In the case of audio-tactile interactions, connections between somatosensory cortex and auditory cortex have been described in several species (Cappe and Barone, 2005; Budinger et al., 2006; Hackett et al., 2007a; Ro et al., 2013; Meredith and Allman, 2015; Godenzini et al., 2021). This is illustrated in Figure 6 for studies carried out in humans (Figure 6A) and in gerbils (Figure 6B). It remains to be determined whether connections from somatosensory cortex to auditory cortical areas, including A1, are causally involved in the multisensory interactions observed physiologically. However, Godenzini et al. (2021) recently reported that photoinhibition of the auditory cortical projection to the forepaw region of the primary somatosensory cortex (S1) eliminated the sound-induced reduction in reaction time on a tactile detection task. This finding, along with other studies of cortical circuitry (reviewed by Meijer et al., 2019), indicates that corticocortical connections are likely to play an important role in mediating multisensory influences on perception and behavior.

Convergence of inputs from different sensory modalities is not, however, restricted to the cerebral cortex. Ascending inputs to the auditory cortex from multisensory regions of the thalamus have been described (Budinger et al., 2006; Hackett et al., 2007b; Cappe et al., 2009; Lohse et al., 2021), indicating that the multisensory properties of cortical neurons may, at least in part, simply reflect their thalamic inputs. In the following section, we briefly review the evidence for somatosensory inputs at different subcortical levels of the central auditory pathway, and outline the circuitry by which tactile stimulation can influence auditory processing.

### Somatosensory influences on subcortical auditory processing

Compared to other sensory systems, the auditory pathway includes a large number of subcortical nuclei, most of which also receive non-auditory inputs (Wu et al., 2015). The first relay in the auditory pathway is the cochlear nucleus. Neurons in the dorsal cochlear nucleus (DCN) are thought to represent spectral localization cues (Yu and Young, 2000), which are primarily responsible for vertical localization and for distinguishing between sound directions in front of and behind the head (Kumpik and King, 2019). This role in sound localization is aided by proprioceptive inputs to the DCN, which in cats provide information about the orientation of the mobile external ears (Kanold and Young, 2001) and in rats may help to distinguish moving sound sources from the apparent movement produced by motion of the head (Wigderson et al., 2016). Somatosensory inputs to the DCN have also been implicated in suppressing the effects of self-generated noises on the central auditory system, such as those produced by vocalizing, licking and masticating (Shore and Zhou, 2006; Singla et al., 2017).

Recently, Ansorge et al. (2021) reported that whisker stimulation can modify sound-evoked activity at this level of the auditory system, enhancing the activity of DCN neurons and producing more diverse and cell-type specific effects in the ventral cochlear nucleus (VCN). Although the functional consequences of these multisensory facilitatory interactions are unclear, electrical stimulation of the spinal trigeminal nucleus has been found to enhance temporal coding by bushy cells, the principal output neurons of the VCN, which has implications for the subsequent processing of vocal and spatial information (Heeringa et al., 2018).

**FIGURE 7:**
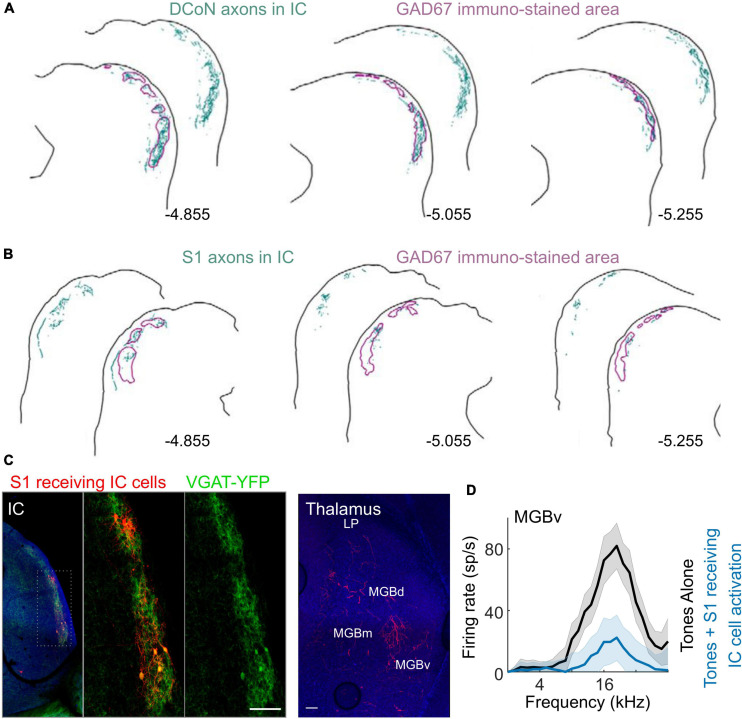
Somatosensory inputs to inhibitory cells in the lateral shell of IC and their influence on auditory responses in the MGBv. **(A)** Axons from the dorsal column nuclei (DCoN) labeled with biotinylated dextran amine (BDA) terminate in inhibitory sectors labeled with GAD67 in the shell of the IC. Labeled axons and terminals are shown in teal, while the outlines of the GAD67 modules are shown in purple. **(B)** BDA-labeled axons from the trunk area of the primary somatosensory cortex (S1) also project primarily to these inhibitory sectors in the shell of the IC. **(C)** Left, anterograde transsynaptic labeling of neurons (red) in the IC shell from S1 (whisker area). These neurons are double labeled with VGAT + (YFP green) and project to the MGBv (right, red axons and terminals). **(D)** Optogenetically activating S1-recipient IC neurons expressing channelrhodopsin inhibits responses to tones in MGBv. LP, lateral posterior thalamus. Panels **(A,B)** adapted from Lesicko et al. (2016). Panels **(C,D)** adapted from Lohse et al. (2021).

Changes in neuronal activity resulting from the integration of auditory and somatosensory signals should also be observed in the downstream targets of the cochlear nucleus. However, Lohse et al. (2021) found no effects of whisker stimulation in mice on the auditory responses of neurons in the central nucleus of the IC (CNIC) (Figure 5), which receives both direct and indirect inputs from the cochlear nucleus. It is possible that some of the somatosensory influences reported in the cochlear nucleus might have subthreshold effects on the activity of CNIC neurons, but this remains to be explored. The CNIC is the core or lemniscal part of the auditory midbrain and is surrounded by a shell comprising the dorsal cortex, a lateral (or external) cortex and a rostral cortex, which can be distinguished by their connections and response properties (Liu et al., 2022). Inputs from multiple subcortical and cortical levels of the somatosensory system have been identified in the IC shell, particularly its lateral cortex (Aitkin et al., 1978; Jain and Shore, 2006; Lesicko et al., 2016; Olthof et al., 2019; Lohse et al., 2021; Figures 7A,B), supporting the notion that the IC should be regarded as a hub for processing auditory signals in the context of other sensory, motor and cognitive information (Gruters and Groh, 2012).

The IC provides most of the auditory input to the superior colliculus, a major site for the integration of multisensory spatial information (Meredith and Stein, 1986; King, 2004). Because spatial information in each sensory modality is encoded using different reference frames, eye position signals have to be incorporated in order to align the different maps of space in the superior colliculus (Jay and Sparks, 1984; Hartline et al., 1995; Populin et al., 2004). The activity of some neurons in both the IC (Groh et al., 2001; Zwiers et al., 2004) and A1 (Werner-Reiss et al., 2003) of monkeys is also affected by eye position. While these signals could arise from proprioceptive feedback from the extraocular muscles, it is more likely that they are conveyed by corollary discharge from brain regions involved in controlling eye movements (Gruters and Groh, 2012).

The IC also projects to the medial geniculate body (MGB) in the thalamus, which provides the gateway to the auditory cortex. Neurons in the medial division of the MGB (MGBm), the suprageniculate nucleus (SGN) and the posterior intralaminar nucleus (PIN) respond to auditory and somatosensory stimuli (Bordi and LeDoux, 1994; Lohse et al., 2021), and, through their projections to the amygdala, are thought to be involved in fear conditioning (Cruikshank et al., 1992; Bordi and LeDoux, 1994). Most of the ascending input from the auditory thalamus to A1 comes from the ventral division of the MGB (MGBv). Although traditionally considered to be a purely auditory structure, somatosensory inputs have been shown to inhibit the activity of neurons in the MGBv (Khorevin, 1980; Kimura and Imbe, 2018; Lohse et al., 2021). Indeed, in mice, concurrent whisker stimulation has been found to suppress the sound-evoked responses of neurons recorded in the MGBv and the adjacent dorsal division of the MGB (MGBd) (Lohse et al., 2021). As with A1, whisker stimulation resulted in divisive scaling of the auditory responses of these neurons and their axon terminals, indicating that these multisensory integrative properties are most likely fed forward to the cortex (Figure 5).

Whisker stimulation has widespread effects on the MGB, suppressing auditory thalamocortical responses and enhancing the auditory responses of neurons in the more medial higher-order thalamic nuclei that principally project to other brain areas. Corresponding effects of audio-tactile stimulation have, however, not been found in the somatosensory thalamus (Lohse et al., 2021). Interactions between other sensory modalities in the rodent thalamus are also asymmetric, since visual stimuli can facilitate the processing of whisker deflection in the ventral posteromedial nucleus, whereas tactile modulation of visual responses has not been observed in the dorsal lateral geniculate nucleus (Allen et al., 2017; Bieler et al., 2018). Together, these findings suggest that multisensory convergence at the level of the thalamus may serve to make objects that touch the whiskers more salient, while reducing the impact of concurrent sounds. Whether this is also the case in primates and other species in which vision dominates multisensory interactions in peripersonal space remains to be seen.

### Circuits underlying somatosensory suppression of auditory cortical activity

**FIGURE 8:**
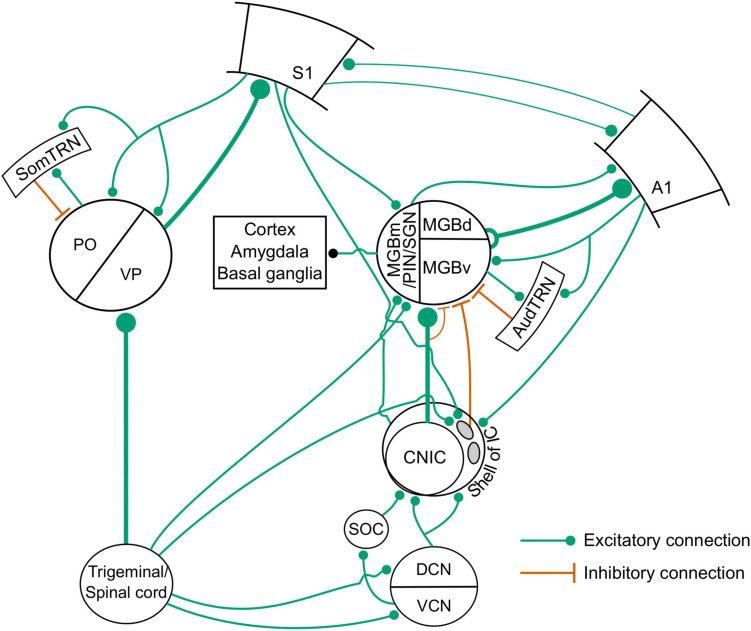
Circuits for somatosensory-auditory interactions in the auditory system. SOC, superior olivary complex; AudTRN, auditory sector of the TRN; SomTRN, somatosensory sector of the TRN; PO, posterior medial nucleus; VP, ventroposterior nucleus. Other abbreviations in main text.

Using a combination of anterograde transsynaptic viral tagging and optogenetic manipulations, Lohse et al. (2021) demonstrated that whisker-stimulation-induced suppression in the auditory thalamocortical pathway is mediated by a corticocolliculo-thalamocortical loop (Figures 7C,D, 8). More specifically, layer 5 neurons in S1 project to a subset of inhibitory neurons in the lateral shell of the IC (Figures 7B,C), which, in turn, project to and suppress the sound-evoked responses of neurons in MGBv and MGBd (Figures 7C,D). The auditory cortex then inherits these suppressed responses from the auditory thalamus. These findings add to a growing body of evidence that communication between different cortical areas can be mediated by transthalamic circuits, as well as by cortico-cortical connections (Lohse et al., 2019; Mo and Sherman, 2019; Figure 8). They also show that the auditory midbrain is a part of the circuitry involved in integrating multisensory signals across the cerebral cortex.

Another structure that could be involved in the modulatory effects of somatosensory stimuli on auditory processing at the level of the thalamus is the thalamic reticular nucleus (TRN), which contains networks of GABAergic neurons that are organized into sensory and motor subdivisions with inhibitory projections to specific thalamic nuclei (Crabtree, 2018; Figure 8). Although previously thought to be modality specific, multisensory convergence has now been demonstrated within the sensory sectors of the TRN, which includes the presence of predominantly suppressive interactions between auditory and somatosensory inputs (Kimura, 2017). However, currently available evidence suggests that the TRN is not part of the circuit implementing the suppressive effects of whisker stimulation on the auditory thalamus (Lohse et al., 2021), though the possibility that this intrathalamic pathway contributes under particular behavioral demands cannot yet be ruled out.

Together, these studies have demonstrated that several neural substrates, involving both ascending and descending projections, exist for combining somatosensory and auditory (as well as visual) inputs in the brain. The somatosensory system can therefore interact with multiple levels of abstraction of the auditory world, from influencing simple frequency representations in the cochlear nucleus to learned relevant auditory categories in the cortex, allowing enormous flexibility in how these circuits operate under different conditions.

## Movement and the auditory system

There is more to understanding the causes, neural underpinnings and functional consequences of multisensory interactions than simply looking at the way particular combinations of sensory stimuli interact to alter the activity of neurons. As we have already stated, sounds are often generated by movement of the body, most obviously in the case of vocalizing, but also during a range of other activities. Indeed, there is growing evidence for a functional role for activation of motor areas in the brain in many aspects of auditory perception, not only for sounds associated with movement—speech, music and other action-related sounds—but even when listening passively (Aglioti and Pazzaglia, 2010; Lima et al., 2016; Froese and González-Grandón, 2020). Furthermore, it has been proposed that unsupervised sensorimotor learning based on the dynamic acoustic inputs resulting from an animal’s own movements can help to establish a stable representation of auditory space in the brain without the need for visual feedback (Aytekin et al., 2008).

**FIGURE 9:**
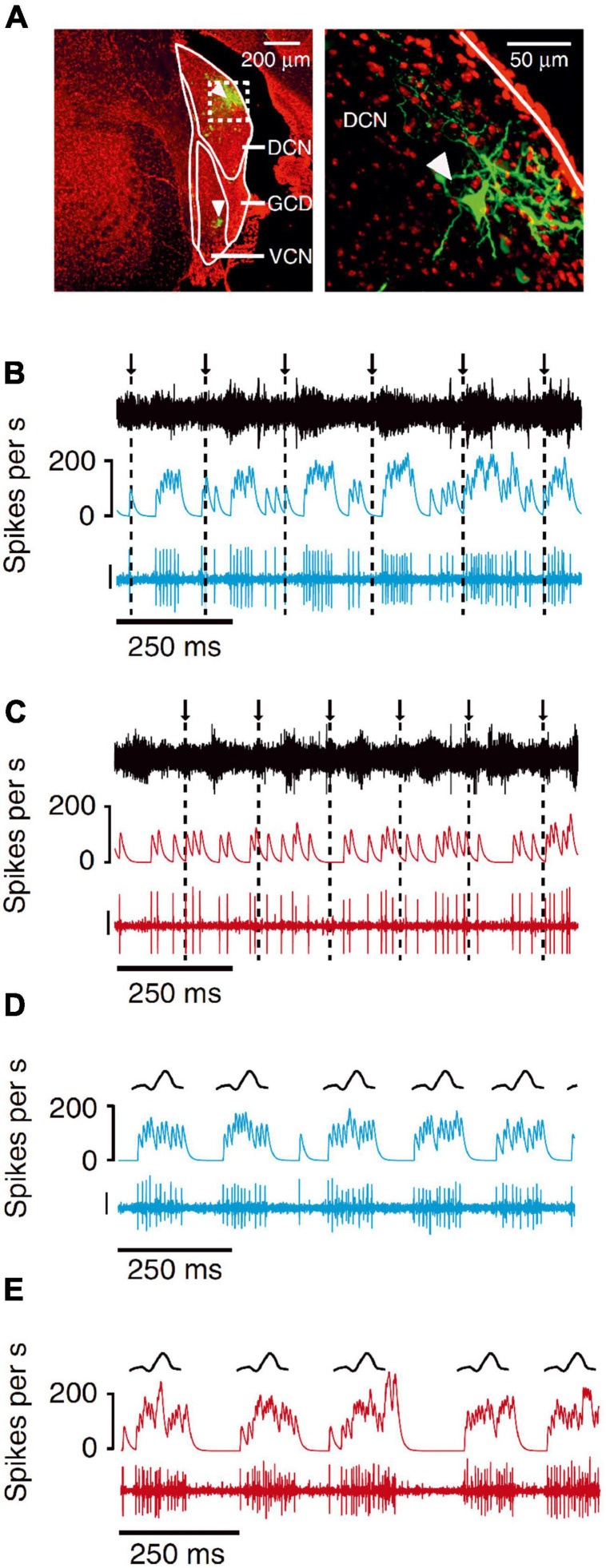
Neurons in the mouse dorsal cochlear nucleus, but not the ventral cochlear nucleus, cancel out the effects of self-generated sounds that result from licking a waterspout. **(A)** Dextran-conjugated Alexa 594 labeling (green) at recording sites in DCN and VCN (arrowheads). DAPI, red. Right, higher magnification of dashed white box on left showing a labeled fusiform cell (arrowhead) in the DCN. **(B)** Example VCN unit exhibiting responses time locked to the licks. Arrows and dashed lines indicate times of tongue contact with the lick spout. Traces represent the microphone recording (top), smoothed firing rate (middle) and VCN unit recording (bottom; scale: 30 μV). **(C)** Example DCN unit recorded during licking. Note the lack of lick-related responses. **(D)** Example VCN unit responses to an externally generated sound with temporal and spectral properties that roughly matched the licking sounds (“lick mimic”). Same unit as in panel **(A)**. Traces represent a schematic of the r.m.s. of the lick mimic (top), smoothed firing rate (middle) and the VCN unit recording (bottom; scale bar: 30 μV). **(E)** Example DCN unit responses to the lick mimic. Same unit as in panel **(C)**. The unit responded to the lick mimic, but not when this sound was generated as a result of the animal’s own licking behavior. Adapted with permission from Singla et al. (2017).

Despite the close relationship between the auditory and motor systems, it is important that the sounds generated by an animal’s own actions are filtered out so that they do not interfere with the processing of auditory stimuli coming from other sources. Vocalizing suppresses auditory cortical activity both in humans (Paus et al., 1996) and animals (Eliades and Wang, 2003). Furthermore, some neurons in auditory cortex respond to perturbed vocal feedback signals (Eliades and Wang, 2008), suggesting the existence of circuits that predict the acoustic consequences of particular movements in order to suppress self-generated auditory inputs. While much of this work has focused on the auditory cortex, as we saw in the section on subcortical pathways, these effects first arise at the level of the cochlear nucleus. Thus, non-auditory inputs allow DCN neurons to cancel out responses to sounds generated by licking movements while retaining their sensitivity to external sounds (Singla et al., 2017; Figure 9). This depends on mossy fiber inputs from the spinal trigeminal nucleus, which presumably convey somatosensory information related to licking behavior.

Modulation of sound-evoked activity is also seen with other movements, including locomotion. In contrast to the facilitatory effect of locomotion on visual cortical responses (Niell and Stryker, 2010), the activity of auditory cortical neurons is typically suppressed, most likely via corollary discharge signals conveyed by the secondary motor cortex to auditory cortical inhibitory neurons (Schneider et al., 2014). This circuit may therefore serve to cancel the predictable acoustic consequences of locomotion and other types of movement (Schneider et al., 2018). The influence of locomotion is not restricted to the auditory cortex, since sound-evoked activity in both the MGB (McGinley et al., 2015; Williamson et al., 2015) and the IC (Yang et al., 2020) is also suppressed when a mouse runs and, at least in the IC, this is accompanied by a sharpening in frequency selectivity (Yang et al., 2020). The source of these suppressive effects on subcortical auditory responses is unclear, but could reflect somatosensory inputs that are activated during locomotion or descending signals from motor cortical areas (Olthof et al., 2019).

**FIGURE 10:**
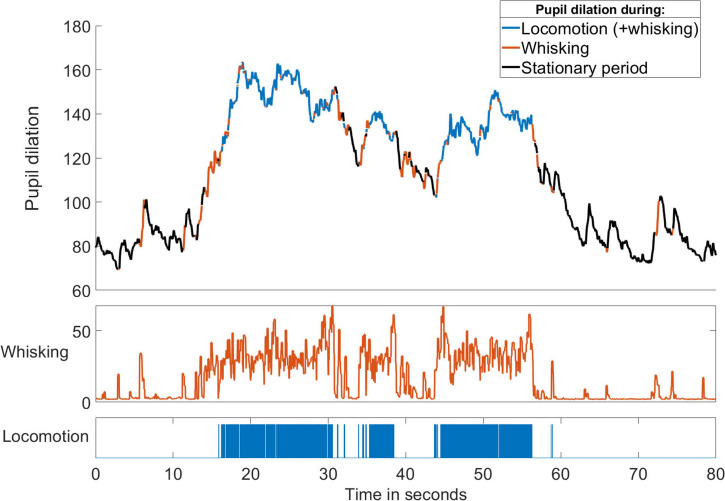
Locomotion and whisking are correlated with brain state. Changes in pupil diameter (in pixels) of a mouse over time with periods of running and whisking indicated by the colors. Increased pupil dilation occurred during running and whisking. Spontaneous whisking often preceded the onset of running and always accompanied locomotion. Whisker velocity was calculated as the maximum movement of a whisker tip in a 30-frame window and was measured as the whisker-tip position change in pixels per frame recorded at 200 fps.

On the other hand, movements tend to co-occur with changes in other physiological parameters (Reimer et al., 2014). Periods of running in rodents usually occur together with eye movements, increasing pupil dilation and rhythmic whisking as the mouse scans the path ahead (Figure 10), an observation consistent with the notion that locomotion is one of several manifestations of a particular brain state and behavioral pattern seen during active exploration. Furthermore, both active whisking (Fanselow and Nicolelis, 1999; Petersen, 2019) and behavioral state (Lee et al., 2020) have been found to affect stimulus processing in the somatosensory cortex, potentially altering its influence on the auditory system. In these circumstances at least, the suppressive effect on auditory responses may not be the result of movement as such, but a consequence of the altered brain state (McGinley et al., 2015) and the purpose of the suppression may not primarily be the cancellation of responses to specific movement-related sounds. Instead, it may reflect a re-allocation of processing resources away from acoustic input and the suppressed auditory cortex toward somatosensory (or visual) cues that provide more useful information about nearby objects while the animal actively explores.

## Conclusion

Although most multisensory research involving audition has focused on its links with vision, extensive interactions also take place between auditory and tactile inputs. This is not restricted to situations where sound-emitting objects approach and eventually touch the body, as in a whining mosquito landing on the back of your neck, but also reflects the fact that sounds are frequently produced during haptic interactions with objects in the environment and as a result of other actions, such as vocalizing. The importance of combining and integrating somatosensory and auditory inputs for perception and the regulation of behavior is indicated by the extensive interactions that have been shown to take place between these two sensory modalities, which have now been demonstrated at almost every level of the auditory system.

We are nonetheless still some way from having a circuit-level understanding of how these modalities interact to influence perception. This largely reflects the few studies that have so far explored audio-tactile interactions at the cellular level in animals trained to perform sensory detection or discrimination tasks. Consequently, relatively little work in non-human species has addressed how attention influences the way these stimuli are integrated and used to guide behavior. In humans, most research on the relationship between attention and multisensory processing has focused on interactions between visual and auditory inputs (Talsma et al., 2010). Nevertheless, selective attention has also been shown to affect the functional coupling between the auditory and somatosensory systems. For example, application of transcranial magnetic stimulation over S1 impaired sound frequency discrimination when human participants simultaneously attended to auditory and tactile frequency, but not when attention was directed to the auditory stimulus alone (Convento et al., 2018). This study also illustrates the value of circuit manipulation methods, which are being used increasingly in humans and animals, for demonstrating a causal contribution of specific brain areas to audio-tactile processing and multisensory integration more generally (Yau et al., 2015).

Details of the neural circuitry involved in merging tactile and auditory signals are still being worked out and it remains unclear why some areas are dominated by facilitatory interactions and others by crossmodal suppression, as is the case, for example, in different divisions of the MGB (Figure 8), or why somatosensory inputs should interface with the auditory system at so many processing levels. One possibility is that tighter regulation of auditory processing may be provided by somatosensory inputs to subcortical structures, whereas cortical involvement may enable greater flexibility crucial for adaptive behavior. The role of some of these circuits may therefore become apparent only under certain conditions (when an animal is engaged in a task, rather than passively listening or anesthetized). Indeed, while the somatosensory system can make us aware of events on the body surface and thus inform us about our immediate surroundings in a passive way, its most important function is to process tactile stimulation during natural behaviors. This means taking brain state into account, utilizing tools for real-time tracking of different parts of the body (Mathis et al., 2018), including individual whisker movements (Sehara et al., 2021), and by considering how different behavioral situations or environmental affordances affect processing (Gibson, 1986), when investigating how somatosensory and auditory inputs are integrated in the brain.

## Author contributions

All authors listed have made a substantial, direct, and intellectual contribution to the work, and approved it for publication.
